# Calculation of Heartbeat Rate and SpO_2_ Parameters Using a Smartphone Camera: Analysis and Testing

**DOI:** 10.3390/s23020737

**Published:** 2023-01-09

**Authors:** Panayiotis Antoniou, Marios Nestoros, Anastasis C. Polycarpou

**Affiliations:** Department of Engineering, University of Nicosia, 2417 Nicosia, Cyprus

**Keywords:** smartphone sensing, heartbeat rate, blood oxygen concentration

## Abstract

Mathematical and signal-processing methods were used to obtain reliable measurements of the heartbeat pulse rate and information on oxygen concentration in the blood using short video recordings of the index finger attached to a smartphone built-in camera. Various types of smartphones were used with different operating systems (e.g., iOS, Android) and capabilities. A range of processing algorithms were applied to the red-green-blue (RGB) component signals, including mean intensity calculation, moving average smoothing, and quadratic filtering based on the Savitzky–Golay filter. Two approaches—gradient and local maximum methods—were used to determine the pulse rate, which provided similar results. A fast Fourier transform was applied to the signal to correlate the signal’s frequency components with the pulse rate. We resolved the signal into its DC and AC components to calculate the ratio-of-ratios of the AC and DC components of the red and green signals, a method which is often used to estimate the oxygen concentration in blood. A series of measurements were performed on healthy human subjects, producing reliable data that compared favorably to benchmark data obtained by commercial and medically approved oximeters. Furthermore, the effect of the video recording duration on the accuracy of the results was investigated.

## 1. Introduction

Recent technological advances in data communication and analysis have provided humankind with immense power and many opportunities. Constant monitoring of biological/vital signals and subsequent rapid analysis by comparison with available data existing all around the world can contribute to improved healthcare. For example, such systems could warn of a coming heart attack or a viral infection. According to a recent report from the WHO, cardiovascular diseases were responsible for an estimated 17.9 million deaths in 2019 [1], of which more than three-quarters were in low- and middle-income countries. As the average life span is increasing, it is very important to limit heart diseases and help people maintain healthy lifestyles. Early detection of abnormalities in vital signals with simple technological tools at a reasonable cost will have a direct economic and social impact. People will be able to monitor their health condition at home or while traveling and could share the information with their physician. Early detection will significantly reduce the financial cost of treating hypertension and subsequent heart conditions (e.g., coronary heart disease) and allow people to be socially active until the end of their lives.

Nowadays, most smartphones are furnished with high-resolution cameras with the possibility of capturing high-definition videos, which can be processed by digital signal-processing (DSP) algorithms to obtain meaningful information hidden in the recorded files. The idea of utilizing smartphones to capture bio-signals has attracted the attention of researchers in various scientific disciplines. Mobile devices have built-in sensors that monitor bio-signals (e.g., sound, pulsation) which carry important information about physiological activities and health status. Furthermore, these signals can be communicated to the user or a healthcare professional through secured communication channels, mobile applications, and cloud services.

The use of the smartphone as a medical sensor device would be extremely useful during major outbreaks of contagious diseases, such as the recent COVID-19 pandemic. In developing countries or remotely populated areas, where access to medical equipment (e.g., oximeters, pulse rate monitors, thermometers, etc.) is very limited, mainly due to cost and availability, smartphones can be used as an alternative device to monitor bio-signals, such as heartbeat rate and oxygen concentration in the blood. During the COVID-19 pandemic, constant monitoring of oxygen concentration levels in the blood was frequently demonstrated to be a life-saving practice. In these cases, access to a smartphone capable of accurately measuring SpO${}_{2}$ and heartbeat rate, using an algorithm embedded in an app could have helped to save millions of lives in poor and developing countries during the recent pandemic. Thus, the aim of this work is to develop dedicated algorithms, for any type of smartphone, able to process bio-signals and produce reliable, accurate and meaningful information concerning heartbeat rate and SpO${}_{2}$.

More specifically, smartphone cameras have been used to capture videos to monitor the pulsation of blood flow through arteries and veins [2,3,4,5,6,7,8,9]. These videos were produced by use of the built-in camera, while the user had their finger firmly attached to the lens. The LED-based flashlight next to the rear camera was used as the light source in this measurement setup. The individual frames of the recorded video were shown to carry information on the pulsating arterial blood flow in the finger. A 30–40 s long video sufficed for the calculation of the pulse rate. The video was then resolved into its frames, which were subsequently split into their three principal color components (red, green, and blue). First, the mean intensity for each frame component was calculated, followed by additional signal processing, which filtered out the noise and provided smoothing of the signal. Hoan et al. [7] describe in detail specific algorithms that were used to detect the local peaks of the pulsating signal and to filter out the noise using a simple moving average filter of seven coefficients. Their results indicated that 87.5% of their cases produced a relative error of less than 5%, whereas, for the remaining 12.5% of cases, the relative error exceeded 5%.

Similar work was undertaken by Bolkhovsky et al. [10] who used the smartphone camera to monitor the index finger, and, hence, to extract the pulse rate using only the green component of the PPG signal. Specifically, these authors used peak detection on the pulsating signal to extract a continuous heart-rate signal. Movement artifacts were manually removed and missed peaks were adjusted accordingly; hence, the approach was not suitable for real-time monitoring. In addition, the video recorded time was between two and nine minutes, which is excessive. The measurements were compared against a five-lead electrocardiogram, showing errors close to 10%.

Lamonaca et al. [11] used an FFT method and a peak-detection approach to extract the pulse rate of the heart. They concluded that the pulse rate, calculated using frequency-domain analysis of the PPG signal, was not comparable with the benchmark value if the video recording time was less than 20 s. Using their proposed peak-detection method, the results compared favorably with the benchmark data (an error of no more than 2 ppm); however, only 10 cases were examined, with pulse rates within the normal range (55–78 ppm).

An interesting approach was described by Lagido et al. [12] who used the smartphone camera to detect patients’ heart rate and rhythm. Specifically, they applied an algorithm based on the most effective methodologies described in the literature to monitor the heart rate of 43 subjects with heart failure. Their experimental results indicated an error of 4.75% compared with data collected from hospital ECGs.

Estimation of the heart rate using recorded videos of the face has also been a subject of research in recent years. Kwon et al. [13] explored the potential of extracting heart-rate data from a distance by recording the face using the front-facing camera of a smartphone. They applied independent component analysis (ICA) to the signal, and then the heart rate was determined using frequency analysis. The duration of the recording was 20 s and there were only 10 participants in the study. Comparison was performed against benchmark heart rates obtained from ECG signals. The reported maximum error was 7.33% for the 10 cases considered in the study.

Holz et al. [8] proposed the use of the front camera using the smartphone’s display light as a source of illumination for the finger. This has potential advantages for the estimation of SpO${}_{2}$ because the narrow-band light emitted from the display in comparison with the broadband light emitted from an LED flashlight results in higher signal contrast between the oxygenated and deoxygenated hemoglobin. The unwanted low frequencies were removed by application of a Gaussian filter with a width of 1 s. Bui et al. [14] recently introduced add-on hardware for the smartphone camera with optical filters, which can isolate specific wavelength bands (green band and red band) emitted by the LED flashlight. In addition, instead of using a traditional linear regression model for the correlation of the SpO${}_{2}$ with the AC/DC signal ratio, an alternative algorithm was used. The results indicated that the approach—together with the add-on device—was able to estimate the blood oxygen saturation within a 3.5% error rate compared to FDA-approved gold-standard pulse oximetry.

Ding et al. [15] used a singular value decomposition (SVD) method to mitigate the effect of large motion artifacts on the accuracy of the signal-processing algorithms. To calculate the oxygen saturation level in blood, they decomposed each color channel into a band-pass (using a Butterworth filter from 0.7 Hz to 4 Hz) and a low-pass component (using a Savitzky–Golay filter: 10 s, order 3). Then, a convolutional neural network (CNN) was applied to improve the SpO${}_{2}$ prediction algorithm. The effect of several parameters, such as the number of convolutional layers, the input window size and the filter length, was investigated to determine the optimum combination. However, the total training time for application of the CNN algorithm was extremely long, rendering the method impractical. The accuracy and repeatability of the measurements are affected by many factors, including the size of the finger and its position over the camera, the stability of the finger during the video-recording process and the illumination level [14,15]. The latter depends on the ambient light as well as the battery status, which might affect the intensity of the emitted light from the LED during the recording. If the pressure level of the finger over the camera is high, the flow of blood through the finger artery is restricted, resulting in miscalculation of the pulse rate.

The purpose of this study was to investigate different methods with respect to their accuracy in predicting heartbeat rate and the ratio-of-ratios by considering various time intervals of video recording. We also considered different filtering methods, controlled by various parameters, which were optimized through parametric studies to provide reliable results. The obtained measurement sets were compared with each other to draw conclusions as to the method providing the most accurate and reliable data, the required recorded video duration, and the impact of the individual video color components on the overall accuracy of the predictions.

## 2. Methodology and Methods of Analysis

**Figure 1:**
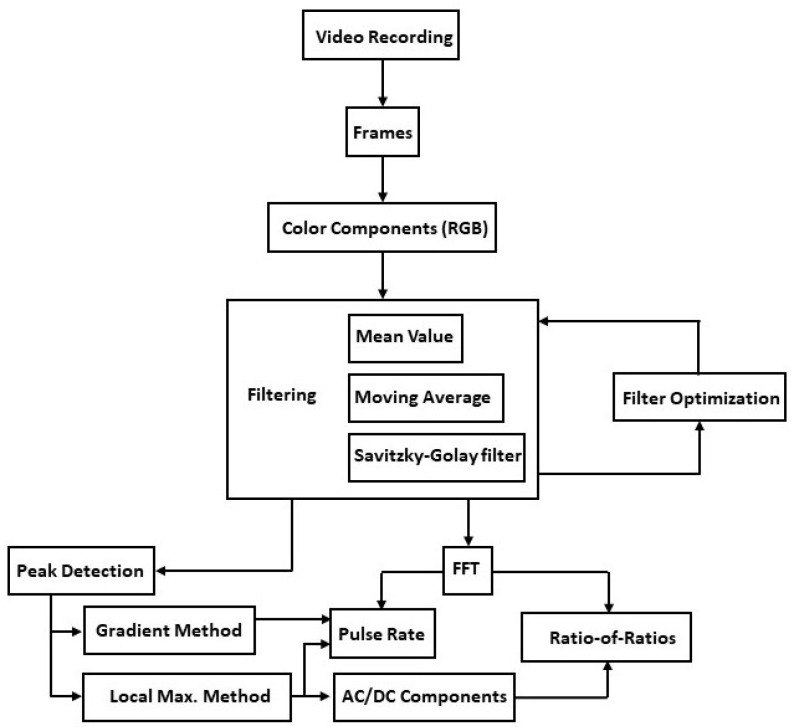
Flowchart of the methodology used to obtain pulse rate and ratio-of-ratios.

RGB component signals obtained from video recording data were processed through a sequence of optimized filtering techniques to deduce vital health parameters. The filtered RGB signals were submitted to two individual scan peak identifiers, namely, the local maximum and gradient methods, which counted the number of local peaks and then calculated the pulse rate. In addition, the analyzed signal, using the local maximum method, was resolved into its DC and AC components to calculate the ratio-of-ratios of the AC and DC components of the red and green signals. Fast Fourier transform was applied to the filtered RGB signals to extract the signal’s dominant frequency component, which correlates to the pulse rate. Furthermore, the FFT method was used to compute the ratio-of-ratios of the red and green signals. The data acquisition and post-processing analysis for the calculation of the pulse rate and the ratio-of-ratios are shown in the flowchart in Figure 1.

The aim of this investigation was to obtain accurate and reliable measurements of the heartbeat pulse rate (PR) and to deduce the oxygen concentration in blood. Consequently, we processed video recordings created by smartphone built-in cameras to extract important health parameters of an individual. Smartphone devices ranging from low-budget ordinary phones to high-tech professional edition smartphones, operating with either Android or iOS operating systems, were used.

A 15 to 45 s long video was recorded from various healthy human test subjects (of varying age, finger skin color, skin volume, fat, bone, etc.). Signal noise caused by background effects, mainly due to the instability of the finger in contact with the camera lens, was mitigated by rejecting 25% of the initial and final parts of the video. All videos were recorded at 30 fps from a rear smartphone camera with the flashlight LED on.

The recorded video was then decomposed into its frames; subsequently, each frame was decomposed into its three principal color components (red, green, and blue). First, the mean intensity for each frame component was calculated, followed by a moving average filter to smooth the measurement data. The moving average filter replaces each data point of a signal with the local average of the surrounding neighboring data points in a sliding window centered at the point. For example, if a given data point has k points to the left and k points to the right, for a total window length of L=2k+1, the moving average filter makes the replacement

(1) $$x_{s}\overset{}{\to }{\hat{x}}_{s}=\frac{1}{L}\sum_{r=-k}^{k}x_{s+r}$$

**Figure 2:**
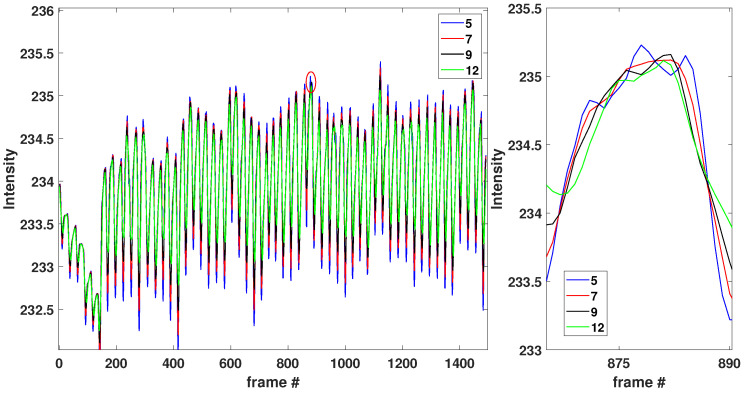
(**Left**): Mean intensity followed by a moving-average filter with several sliding window lengths vs. frame number. (**Right**): shows, in magnification, the region around the peak at frame 880.

Based on our investigation, we concluded that the moving average filter provided a signal with minimum noise when each individual mean was calculated over a sliding window of length seven across neighboring data elements of the initial mean intensity calculated for each frame component. Figure 2 (left) shows the same signal intensity obtained with different moving-average window lengths (e.g., 5, 7, 9, and 12 neighboring data elements window). Figure 2 (right) shows magnified the red circled peak illustrated in Figure 2 (left) at frame 880. Figure 2 (right) shows that a sliding window of length seven across neighboring data elements produced smoother results.

**Figure 3:**
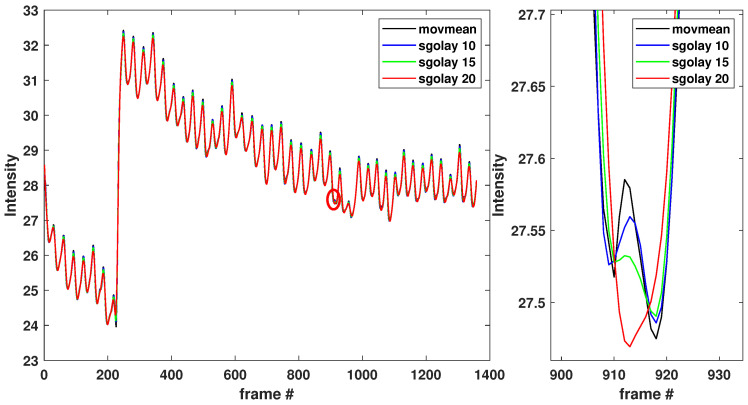
(**Left**): signal intensity obtained with the moving-average filter applied with and without the Savitzky–Golay filter (with several sliding window lengths). The red circled peak at frame 915 is magnified on the right. The red circled peak at frame 915 shows that a sliding window of length 20 across neighboring data elements gives smoother results.

Despite the smoothing effect of the moving-average filter, we still obtained traces of noise caused by several background factors, e.g., high-frequency modes, external light sources, and, possibly, low-quality cameras. We wished to achieve smooth oscillatory signals with distinct local extrema that would enable us to analyze further the signal obtained by any type of smartphone. To this end, after applying the moving-average filter, we applied a Savitzky–Golay filter, which smooths according to a quadratic polynomial that is fitted over each window of the mean intensity obtained by the moving average filter. Our investigation demonstrated that the Savitzky–Golay filter provided a smoothed/denoised signal when each least-squares polynomial fit was calculated over a sliding window of length 20 across neighboring data elements of the mean intensity obtained by the moving-average filter. Figure 3 (left) shows the same signal intensity obtained with different window lengths (e.g., 10, 15, and 20 neighboring data elements window). Figure 3 (right) shows magnified the red circled peak at frame 915. Figure 3 (right) shows that the Savitzky–Golay filter applied with a 20-element sliding window was more effective than other methods when the data varied rapidly.

Developing a neat oscillatory signal leads to efficient and accurate calculation of the heartbeat pulse rate. The calculation of the pulse rate was performed with two equivalent methods that gave identical results. The main purpose of the use of both methods was to identify the peaks in the signal and to count their number in each window. Knowing the number of peaks and the time length of the window, the pulse rate can be readily estimated. For the first method (the local maximum method), we identified the peaks as local maximums; in the second method (the gradient method), the peaks were identified by calculating the gradient at nearby points to the left and right of the evaluation points of interest. A particular data point was identified as a peak (e.g., a maximum) if the gradient was positive at three consecutive points before the peak and negative at three consecutive points following the peak.

**Figure 4:**
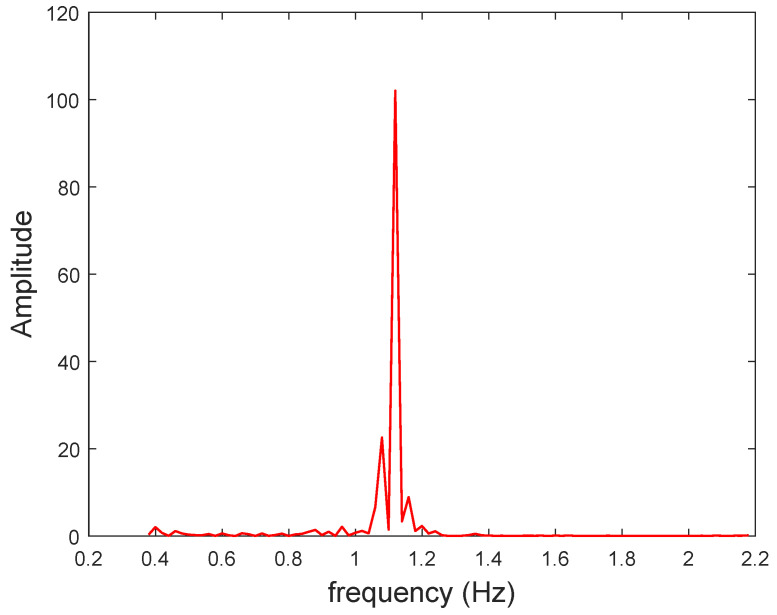
Typical FFT spectrum from a smoothed mean intensity signal (red channel). The estimated pulse rate is 67 bpm (1.1195 Hz).

The smoothed mean intensity signal was also processed by fast Fourier transformation (FFT) to correlate the frequency components of the signal with the pulse rate (FFT method). The amplitude of the signal in the frequency domain produced peaks in the 0.5–2 Hz range (Figure 4). The frequency spectrum exhibited a main peak, with maximum amplitude, the frequency of which approximated the heart pulse rate. A series of experiments were performed with *N* healthy subjects, with ages ranging from 17 to 82 years. The peak frequency $f_{p}$ in Hz was multiplied by 60, to obtain the pulse rate. The results for this method were compared with the benchmark reading (using a medically approved oximeter) and the other two aforementioned methods. The relative deviations of the three methods are tabulated below.

**Figure 5:**
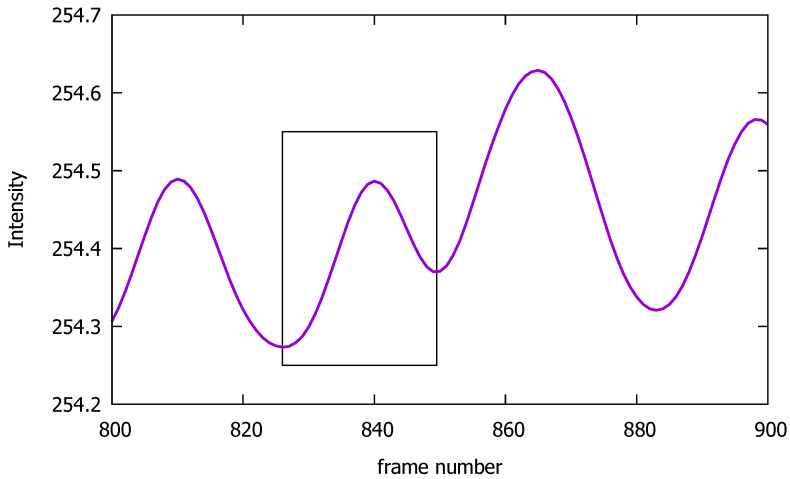
Schematic diagram representing a random frame window used to calculate the AC and DC components of the signal.

We further decomposed the signal into its AC and DC components. We split the signal into frame windows (Figure 5) with each frame window limited between two consecutive local minimums. The DC component of each individual frame window was defined as the average value of the signal in the range between the two local minimums. The AC component magnitude was defined to have the value of the intervening local maximum between the two local minimums of the respective frame window.

In our numerical code, we developed a sliding frame window that deduced all the AC and DC components for the red and green spectral components of the signal. The blue component of the signal, as is illustrated in the Results section, was excluded from our study because of its low correlation with the heartbeat pulse rate. The oxygen concentration in blood can, in principle, be calculated in a non-invasive way using the photo-plethysmography technique PPG [6,14], which is based on the differential absorption of light of two different wavelengths from oxygenated and de-oxygenated arterial blood.

According to the theoretical treatment of the PPG technique, the oxygen concentration in blood SpO${}_{2}$ can be derived from the ratio-of-ratios *R* of the AC and DC components, calculated at two different wavelengths $\lambda_{1}$ and $\lambda_{2}$ around an isosbestic point. A commercial fingertip pulse oximeter generally uses two LEDs, generating red (approx. 650 nm) and infrared light (approx. 950 nm), as well as photo detectors. In this study, the red and green components (broadband) of the recorded video signals were used instead.

(2) $$R=\frac{{\left(AC/DC\right)}_{\lambda_{1}}}{{\left(AC/DC\right)}_{\lambda_{2}}}$$

SpO${}_{2}$ is usually determined by linear or quadratic regression analysis. In these models, the ratio-of-ratios R, as given in the above expression, is the independent variable directly related to blood oxygenation [4,6].

## 3. Results

A group of 35 volunteers (aged between 17 and 82 years) participated in the testing phase. Specifically, the PR and blood oxygenation (SpO${}_{2}$) were measured using medically approved oximeters simultaneously with capture of the video of the index finger directly attached to the smartphone camera. The video recordings were made for three different time intervals: 15 s, 30 s, and 45 s. In the following, we discuss the accuracy of the three methods implemented for the calculation of PR, the impact of the recording time duration on the results, and the calculation of the ratio-of-ratios, used for the prediction of SpO${}_{2}$, based on two methods.

### Comparison of the Various Methods for the Heartbeat Rate Calculation

As mentioned in the previous section, the heartbeat rate was calculated based on three methods: (a) the gradient method; (b) the local maximum method (available as a ready-made function by Matlab) and, (c) the FFT method. To evaluate the accuracy of each method, the calculated PR for each individual component of the RGB video signal was normalized against the benchmark reading obtained from a medically approved oximeter. Specifically, we used Braun and Beurer oximeters [16]. The video recordings were obtained by three different smartphones: an iPhone 8 (iOS 16.11), a Samsung Galaxy A22 5G (Android 12) and a Xiaomi Redmi 9A (Android 10). The reason we used different types of smartphones, as well as oximeters, was to generate unbiased data that would be insensitive to a specific brand or operating system.

The recording of the videos was performed by different individuals using the described smartphones and oximeters. The volunteers who participated in the study placed their left index finger in the oximeter, used as a benchmark, and their right index finger was attached directly to the smartphone rear camera with the flashlight always on during the measurement. The durations of recording were 15 s, 30 s and 45 s. This was performed to evaluate the impact of the time duration of the video on the accuracy of the results. It is worth noting that the flashlight required always to remain on during the recording, otherwise the pulsating signal would be superseded by interference from surrounding light sources—the main spectral component from the FFT response would then not be clearly identified from other spectral components caused by other light sources. The same observation applied to the other two methods, where the pulsating response due to the actual heartbeat was extremely weak and unidentified.

**Figure 6:**
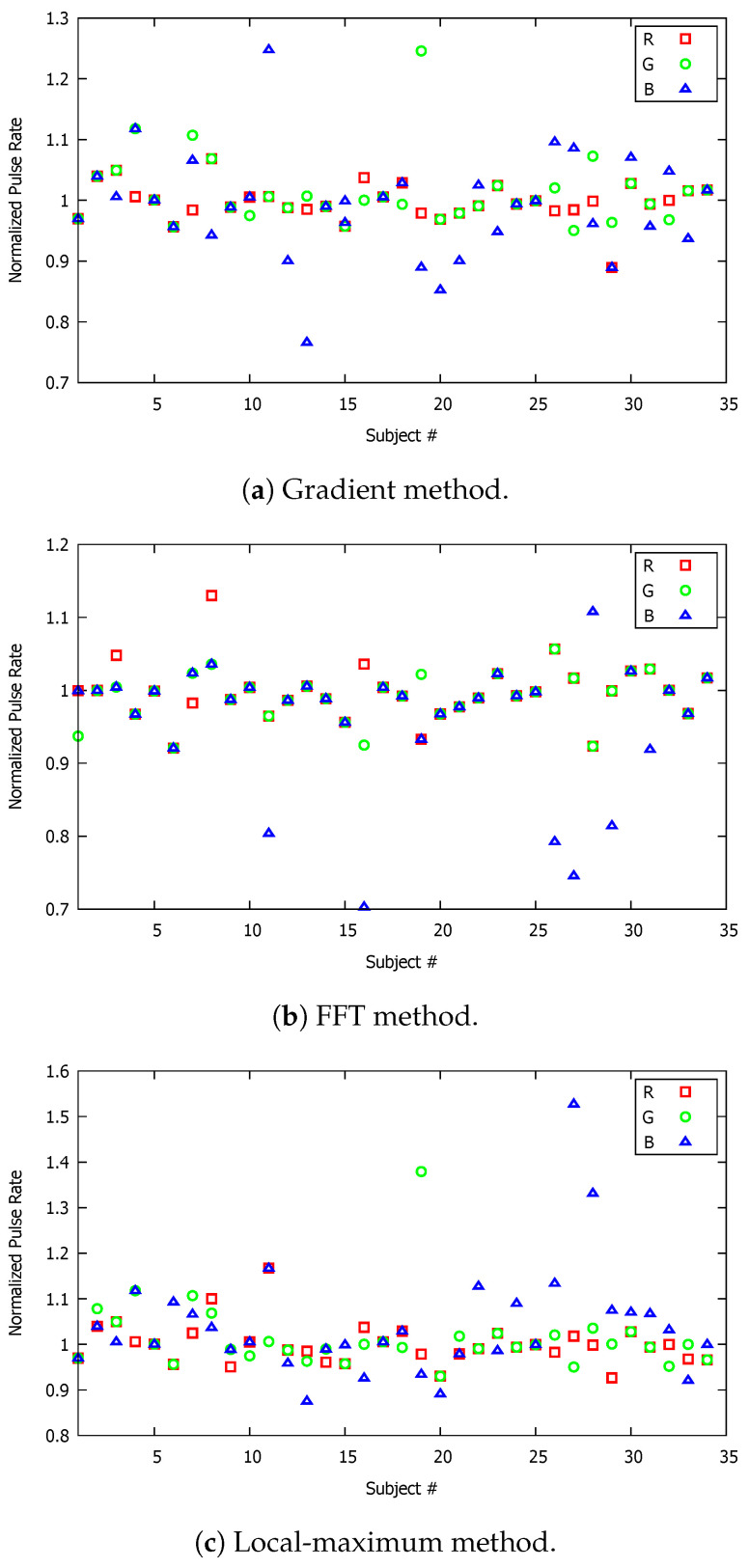
Normalized pulse rate (PR) of all participating volunteers; the video recording time in these graphs corresponds to 45 s.

The first set of measurement data taken by four independent researchers, trained to the agreed methodology, corresponded to a recording time duration of 45 s. The recorded videos were analyzed, based on the three previously discussed techniques, using MATLAB. In Figure 6a, we present the corresponding measurements for the heartbeat rate, normalized by the average benchmark reading obtained by the oximeter. It is of note that the oximeter reading, though obtained by a medically approved device, is susceptible to a small degree of error due to possible movement/mispositioning of the finger or interference by other nearby sources. In addition, the oximeter’s device manufacturer’s stated accuracy is +/− 1 bpm, for less than or equal to 100 bpm, and +/− 2 bpm for greater than 100 bpm. The results for application of the gradient method with corresponding normalized measurements for 45 s are shown in Figure 6a. In this graph, three different color markers correspond to the three color components of the recorded video. It is clearly shown that the red and green components were less dispersed from the benchmark average, which was unity, indicating their increased accuracy compared to the blue component prediction of the heartbeat rate. This can be also observed from Table 1, which tabulates the average value along with the standard deviation of the analyzed results. Specifically, the average value obtained for the three video channels, i.e. red, green and blue, were 1.00, 1.01 and 1.01, respectively. The corresponding values of the standard deviation were 0.024, 0.040, and 0.096, clearly indicating that the analyzed results based on the blue component were much more dispersed compared to the other two components, though this was not quite as evident from the average values, which differed by less than 1%. As a result, the gradient method, as applied to the 45-s video recordings, provided more reliable and accurate PR predictions for the red and the green channels.

The FFT and local maximum methods were also applied to the same measurement dataset. The results of the analysis are shown in Figure 6b,c, respectively. Clearly, the red and the green channels provided better prediction of the heartbeat rate compared to the blue channel. As seen from Figure 6b, which corresponds to the FFT method, the blue markers were more dispersed from the average benchmark reading compared to the other two color markers. Specifically, the mean values for the red and the green channels were 1.00 and 0.99, respectively, whereas, for the blue channel, the mean value was 0.95, indicating a larger deviation from the benchmark reading. The same inference follows for examination of the results for the standard deviation, which was 0.107 for the blue, and 0.032 and 0.034 for the red and green channels, respectively. For the local maximum method, the results were quite similar to those for the other two methods, indicating more reliable and accurate results for the red and green channels compared to the blue channel. More specifically, according to Table 1, the average readings for the three channels were 1.01, 1.01 and 1.07, for the red, green and blue channels, respectively. The corresponding standard deviation values were 0.046, 0.042 and 0.152, respectively. It can be inferred that the results from the blue channel were less accurate than for the other two colour channels for the heartbeat rate.

**Figure 7:**
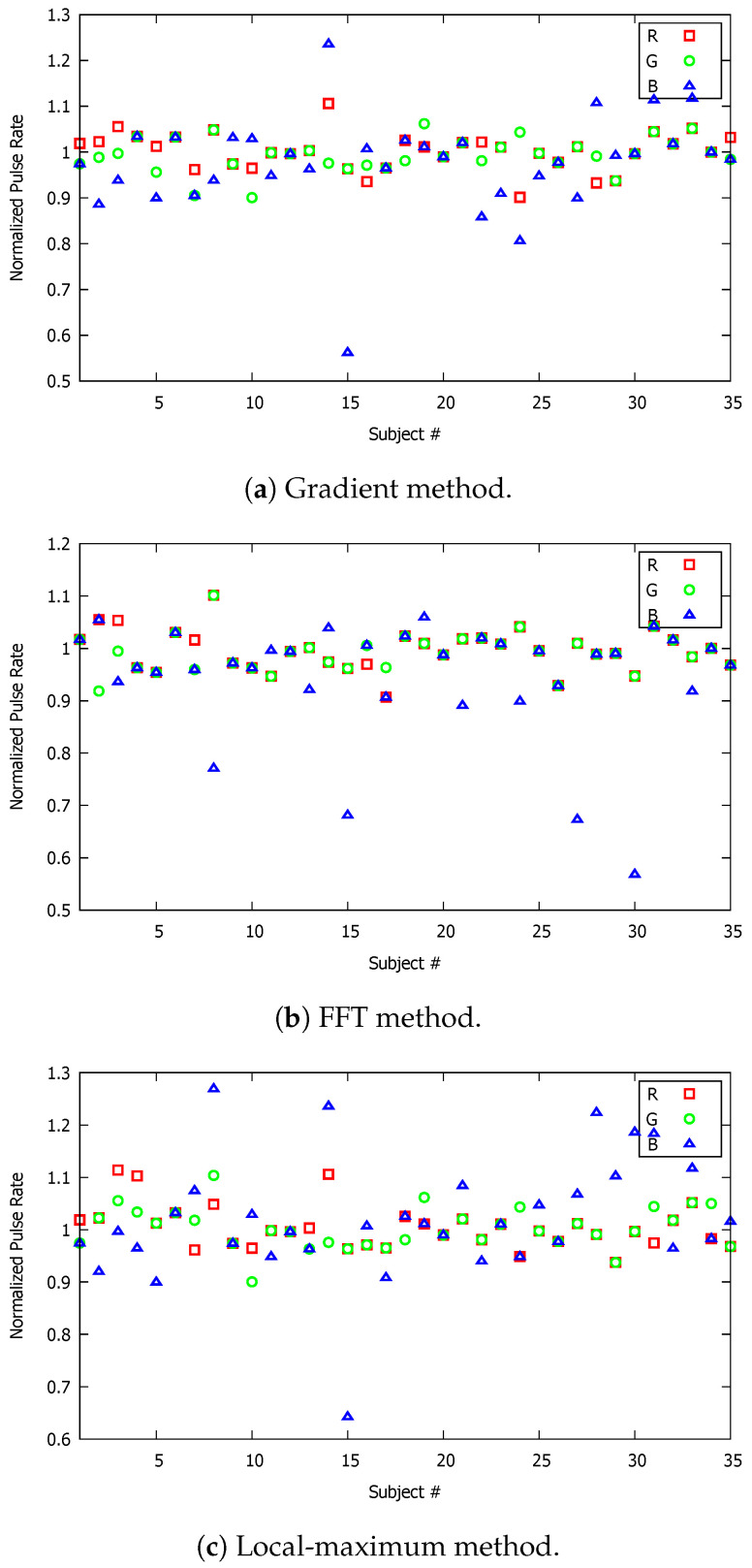
Normalized pulse rate (PR) of all participating volunteers; the video recording time in these graphs corresponds to 30 s.

**Figure 8:**
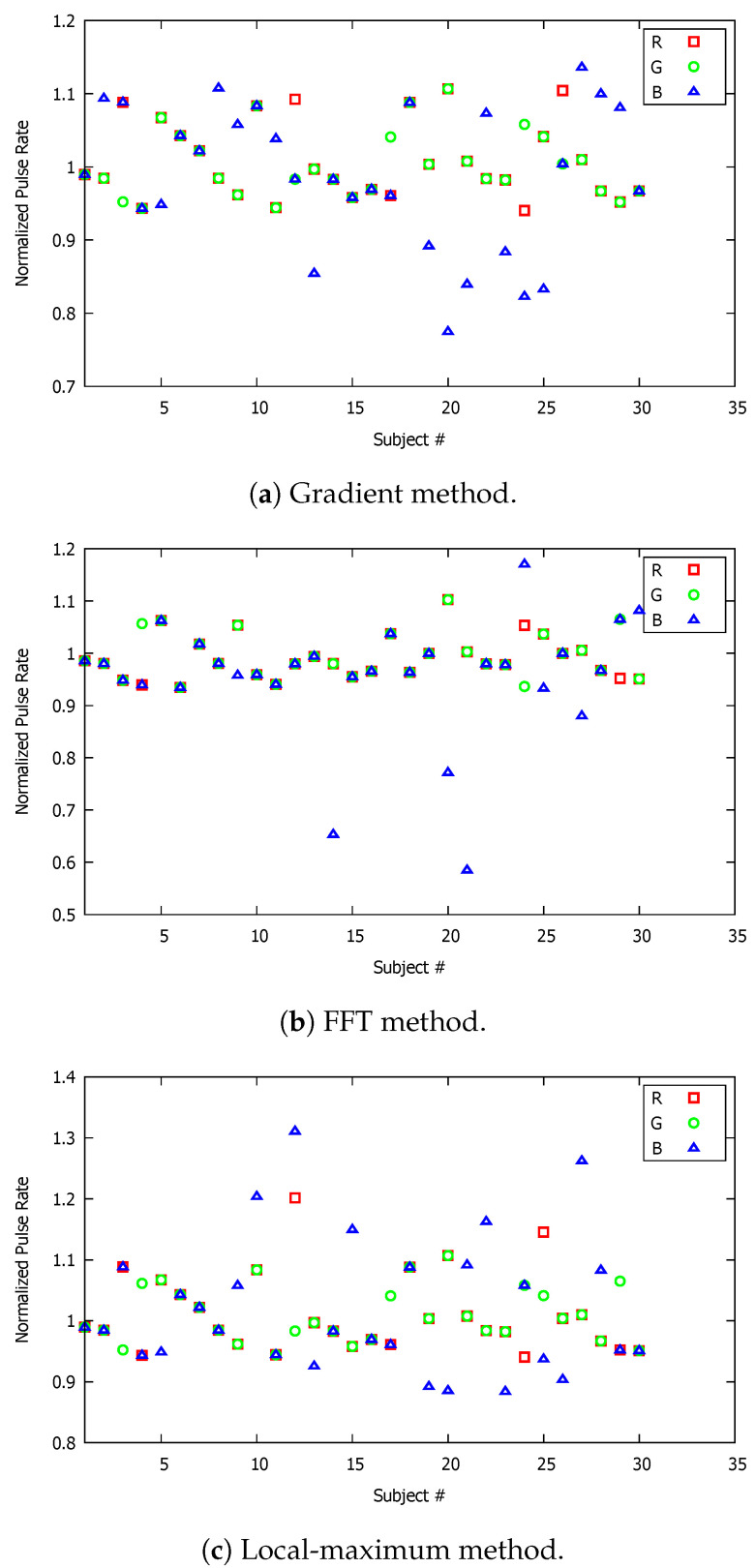
Normalized pulse rate (PR) of all participating volunteers; the video recording time in these graphs corresponds to 15 s.

The experiment was repeated for a shorter duration of video recordings. Specifically, we conducted the same type of measurements for a video recording duration of 30 s, and, subsequently, for 15 s. We reduced the video recording time, initially from 45 s to 30 s and then from 30 s to 15 s to investigate the video duration effect on the accuracy and reliability of the pulse-rate measurements using the smartphone camera. It is of critical importance to identify an acceptable (preferably as short as possible) video recording time interval for which the accuracy of the measurements is not compromised. It is important to emphasize that the experiments conducted for the three different recording times were independent of each other, meaning that the procedure was repeated as if it was a new experiment. In addition, the participating volunteer for each case may not have been the same individual for all three recording time intervals. The corresponding measurements obtained are shown graphically in Figure 7 and Figure 8 for 30 and 15 s, respectively. At first glance, it is evident that the blue triangular markers, which correspond to the blue component of the RGB signal, are more distant from the benchmark reading. The PR measurements based on the red and green components were closely concentrated around the benchmark value. This also applied for the 45 s video recording time. If the average values based on each individual color component are compared for each of the three implemented approaches (see Table 1, Table 2 and Table 3), it can be concluded that the video recording duration had no significant effect on the accuracy of the methods. This was also evident by comparing the standard deviation of each method for the three video recording time intervals. This represents surprisingly good news, as all three methods can provide accurate and reliable PR results by video recording of the index finger for a short duration of only 15 s.

**Figure 9:**
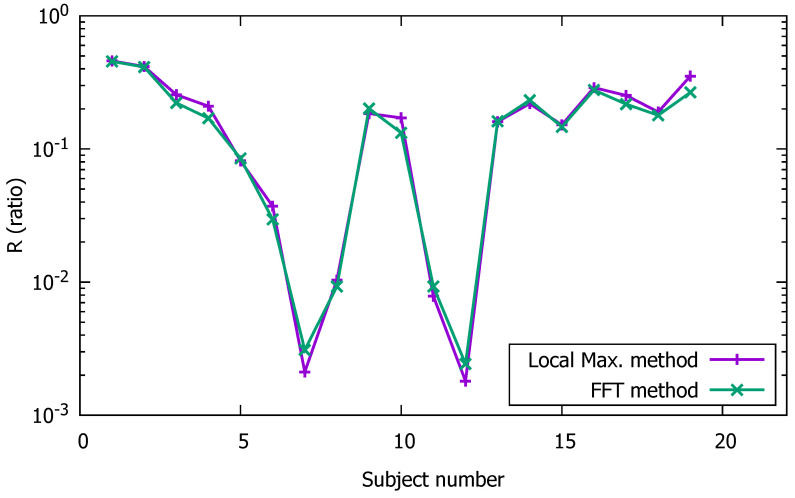
Ratio-of-ratios for a group of 19 healthy individuals calculated using the FFT method and the local maximum method.

In addition to monitoring the heartbeat rate of an individual by use of a smartphone camera, our initial objectives also included monitoring the SpO${}_{2}$. To calculate the percentage of blood oxygenation using pulse oximetry, it is first necessary to estimate the ratio-of-ratios for two colors, as defined in (2). Then, a linear or quadratic regression formula with calibrated coefficients is used to estimate SpO${}_{2}$. Therefore, our goal was to calculate the ratio-of-ratios for the red and green color components of the video signal, and, then, to examine its behavior for different types of phones and participating individuals. All the participants in this type of measurement had a percentage blood oxygenation between 95% to 99% for the entire duration of the experiment. In other words, the SpO${}_{2}$ reading of the commercial oximeter varied by no more than 5% for all measurements. As shown though in Figure 9, the calculated value of the ratio-of-ratios, using the FFT method and the local maximum method, varied significantly from individual to individual and from one phone type to another. Figure 9 shows 19 different measurements, which correspond to 19 healthy volunteers, and three different types of smartphone. As can be clearly seen, the variation in the ratio-of-ratios was close to an order of magnitude of three, even though the benchmark percentage blood oxygenation (obtained by the commercial oximeter) did not vary by more than 5%. For this reason, we did not implement this type of approach for the estimation of SpO${}_{2}$, mainly due to the lack of correlation between the ratio-of-ratios and the actual oxygen concentration level in the blood of an individual. This weakness in the current methodology can be mainly attributed to the significant variation in the light intensity of the flashlight during the course of the measurements, in addition to key hardware differences and technical specifications for the different types of smartphone used in this investigation. In this study, it was also observed that the intensity of the flashlight was closely correlated to the smartphone’s battery charge state, which might have affected the accuracy of the calculation of the ratio-of-ratios for the subjects participating in this research. In future investigations, we will seek to isolate these technical parameters from the SpO${}_{2}$ calculation by incorporating the same type of smartphone for all measurements, while constantly monitoring the state of the battery.

## 4. Conclusions

In this study, we investigated three different methods, along with different types of filtering techniques, for estimating the heartbeat rate of individuals using solely a smartphone camera to record a short video of the index finger attached directly to the camera. The analysis was based on the three color components of the video signal. All three methods incorporating optimized filtering techniques provided fairly good results compared to a benchmark reading obtained from a medically approved oximeter available commercially. Analysis of the three color components clearly showed that the predictions of the heartbeat rate for the red and green color components were more accurate than that for the blue component. The video recording duration was also found not to affect the accuracy of the pulse-rate calculation. We demonstrated that a 15 s recorded video was adequate for accurate and reliable estimation of the pulse rate based on the red and green color components. Two of these methods were applied for estimation of the SpO${}_{2}$ by first calculating the ratio-of-ratios, which is widely discussed in the literature. However, our calculations showed that the obtained ratio did not correlate with the oxygen concentration reading of a commercial oximeter; thus, we did not proceed with the regression analysis. Future investigations are needed to ensure a reliable approach to the estimation of SpO${}_{2}$ using a smartphone camera. 

## Figures and Tables

**Table 1:** Statistical data for 45-s video recordings.

Color Component (Method)	Average	Standard Deviation
Red (Gradient)	1.00	0.024
Green (Gradient)	1.01	0.040
Blue (Gradient)	1.01	0.096
Red (FFT)	1.00	0.032
Green (FFT)	0.99	0.034
Blue (FFT)	0.95	0.107
Red (Local max)	1.01	0.046
Green (Local max)	1.01	0.042
Blue (Local max)	1.07	0.152

**Table 2:** Statistical data for 30-s video recordings.

Color Component (Method)	Average	Standard Deviation
Red (Gradient)	1.00	0.045
Green (Gradient)	0.99	0.034
Blue (Gradient)	0.95	0.124
Red (FFT)	1.00	0.045
Green (FFT)	0.99	0.041
Blue (FFT)	0.92	0.132
Red (Local max)	1.01	0.049
Green (Local max)	1.00	0.038
Blue (Local max)	1.02	0.137

**Table 3:** Statistical data for 15-s video recordings.

Color Component (Method)	Average	Standard Deviation
Red (Gradient)	1.00	0.050
Green (Gradient)	0.99	0.038
Blue (Gradient)	0.97	0.089
Red (FFT)	0.98	0.038
Green (FFT)	0.98	0.038
Blue (FFT)	0.94	0.143
Red (Local max)	1.01	0.069
Green (Local max)	1.00	0.040
Blue (Local max)	1.03	0.113

## Data Availability

Not applicable.
